# The Association of Dietary Intake of Purine-Rich Vegetables, Sugar-Sweetened Beverages and Dairy with Plasma Urate, in a Cross-Sectional Study

**DOI:** 10.1371/journal.pone.0038123

**Published:** 2012-06-06

**Authors:** Lina Zgaga, Evropi Theodoratou, Janet Kyle, Susan M. Farrington, Felix Agakov, Albert Tenesa, Marion Walker, Geraldine McNeill, Alan F. Wright, Igor Rudan, Malcolm G. Dunlop, Harry Campbell

**Affiliations:** 1 Centre for Population Health Sciences, University of Edinburgh, Edinburgh, United Kingdom; 2 Andrija Stampar School of Public Health, Medical School, University of Zagreb, Zagreb, Croatia; 3 Colon Cancer Genetics Group and Academic Coloproctology, Institute of Genetics and Molecular Medicine, University of Edinburgh and MRC Human Genetics Unit Western General Hospital, Edinburgh, United Kingdom; 4 Public Health Nutrition Research Group, The Rowett Institute of Nutrition and Health, Greenburn Road, Bucksburn, Aberdeen, United Kingdom; 5 Pharmatics Limited, Edinburgh, United Kingdom; 6 MRC Human Genetics Unit; Institute of Genetics and Molecular Medicine, Western General Hospital, Edinburgh, Scotland, United Kingdom; Wageningen University, The Netherlands

## Abstract

**Introduction:**

Hyperuricemia is a strong risk factor for gout. The incidence of gout and hyperuricemia has increased recently, which is thought to be, in part, due to changes in diet and lifestyle. Objective of this study was to investigate the association between plasma urate concentration and: a) food items: dairy, sugar-sweetened beverages (SSB) and purine-rich vegetables; b) related nutrients: lactose, calcium and fructose.

**Methods:**

A total of 2,076 healthy participants (44% female) from a population-based case-control study in Scotland (1999–2006) were included in this study. Dietary data was collected using a semi-quantitative food frequency questionnaire (FFQ). Nutrient intake was calculated using FFQ and composition of foods information. Urate concentration was measured in plasma.

**Results:**

Mean urate concentration was 283.8±72.1 mmol/dL (females: 260.1±68.9 mmol/dL and males: 302.3±69.2 mmol/dL). Using multivariate regression analysis we found that dairy, calcium and lactose intakes were inversely associated with urate (p = 0.008, p = 0.003, p = 0.0007, respectively). Overall SSB consumption was positively associated with urate (p = 0.008), however, energy-adjusted fructose intake was not associated with urate (p = 0.66). The intake of purine-rich vegetables was not associated to plasma urate (p = 0.38).

**Conclusions:**

Our results suggest that limiting purine-rich vegetables intake for lowering plasma urate may be ineffectual, despite current recommendations. Although a positive association between plasma urate and SSB consumption was found, there was no association with fructose intake, suggesting that fructose is not the causal agent underlying the SSB-urate association. The abundant evidence supporting the inverse association between plasma urate concentration and dairy consumption should be reflected in dietary guidelines for hyperuricemic individuals and gout patients. Further research is needed to establish which nutrients and food products influence plasma urate concentration, to inform the development of evidence-based dietary guidelines.

## Introduction

The incidence rate of gout has increased substantially over recent years [1], [2] in parallel with a rising prevalence of hyperuricemia [3]. Possible underlying reasons include recent changes in diet, lifestyle and increasing prevalence of obesity. Understanding the determinants of plasma urate concentration is relevant for the successful management of gout. Additionally, hyperuricemia has been associated with metabolic syndrome and implicated as a risk factor in the etiology of hypertension, atherosclerosis, insulin resistance and diabetes [3], [4].

The association between a purine-rich diet and an increased plasma urate concentration and risk of gout has long been recognized. Uric acid is the end-product of purine degradation so the avoidance of purine-rich foods is commonly recommended to gout patients [5], [6]. Studies have also reported an association between the intake of sugar-sweetened beverages (SSB) and urate concentration and gout [7], [8], [9]. In contrast, it has been shown that some foods are inversely associated with plasma urate concentration, primarily low-fat dairy products and foods rich in vitamin C [8]. However, sugar-sweetened beverages are rarely included in the gout dietary guidelines.

Several aspects of the relationship between diet and hyperuricaemia and gout are uncertain and thus the implications for dietary recommendations for those are currently unclear. A large prospective study of gout comprising 47,150 men failed to show an association between consumption of purine-rich vegetables and risk of gout [10]. This finding challenged the current recommendation to restrict purine-rich vegetable intake in gout patients. Next, fructose has been proposed as the causal agent underlying the association between SSB and urate, but it is unclear whether the amount of fructose found in SSB, or in the diet, is sufficient to account for this.

An accurate understanding of which food products and which nutrients affect plasma urate concentration is important if effective evidence-based dietary recommendations are to be formulated. Inappropriate assumptions could lead to unnecessary and potentially harmful dietary restrictions (e.g restriction of purine-rich vegetables due to suggested urate-increasing effect). Current recommendations include restriction on alcohol, meat and other purine-rich foods, but formulating recommendations regarding dairy products and SSBs should also be considered [11].

In this study, we set out to test the reported association between plasma urate concentration and the intake of purine-rich vegetables, sugar-sweetened beverages and dairy.

## Results

We investigated plasma urate concentrations of 2,037 healthy individuals (44% female), mean age: 62 (10.5) years, range: 21–82 y. The mean urate concentration in this population was 283.79 (72.12) mmol/dL (women: 260.06 (68.85) mmol/dL, men: 302.28 (69.17) mmol/dL). In our sample 11.3% of female and 5.2% of male participants were hyperuricemic (cut-off values of >415 mmol/dL for men and more stringent >340 mmol/dL for women). Some characteristics of the study population according to urate concentration tertiles are shown in **Table 1**.

**Table 1:** Characteristics of our Cohort According to Urate Concentration, Healthy Adults From Scotland, UK (1999–2006).

	all	<250 mmol/dL	250–310 mmol/dL	>310 mmol/dL
**N all**	2037	742 (35.74%)	561 (27.02%)	734 (35.36%)
**N female (% female)**	892 (43.7%)	462 (51.79%)	224 (25.11%)	206 (23.09%)
**Age, ** ***y***	62 (10.5)	59.9 (10.7)	62.8 (10.4)	63.5 (10)
**Urate, ** ***mmol/dL***	283.8 (72.1)	212.3 (31.8)	279.3 (14.4)	359.5 (48.4)
**BMI**	26.8 (4.7)	26 (4.6)	27.1 (5)	27.5 (4.6)
**Energy, ** ***MJ***	10.9 (4)	10.6 (3.8)	11 (4.3)	11.1 (4)
**Dairy, ** ***25 g portions/day***	13.4 (8.2)	14 (8.7)	13.6 (8.1)	12.8 (7.8)
**Meat, ** ***servings/day***	2.5 (1.6)	2.4 (1.4)	2.6 (1.7)	2.7 (1.6)
**Seafood, ** ***servings/day***	0.9 (0.8)	0.9 (0.8)	0.9 (0.8)	0.9 (0.9)
**Purine-rich vegetables, ** ***tablespoons/day***	1.5 (1.2)	1.5 (1.2)	1.5 (1.1)	1.5 (1.2)
**Fizzy drinks, ** ***servings/day***	0.1 (0.3)	0.1 (0.3)	0.1 (0.2)	0.1 (0.4)
**Squashes, ** ***servings/day***	0.3 (0.8)	0.3 (0.8)	0.2 (0.6)	0.3 (0.8)
**Pure fruit juice, ** ***servings/day***	0.7 (0.9)	0.7 (0.8)	0.7 (0.9)	0.7 (0.9)
**Low-calorie fizzy drinks, ** ***servings/day***	0.3 (0.7)	0.3 (0.7)	0.3 (0.8)	0.3 (0.7)
**Coffee, ** ***servings/day***	1.8 (1.8)	1.9 (1.9)	1.7 (1.8)	1.7 (1.7)
**Alcohol, ** ***g/day***	8.7 (12.1)	6.8 (9.4)	8.5 (12.8)	11.1 (13.5)
**SSB, ** ***servings/day***	0.4 (0.8)	0.3 (0.9)	0.3 (0.7)	0.4 (0.9)
**Vitamin C, ** ***mg/day***	157.8 (148.4)	177.9 (169.7)	151.1 (141.4)	143.8 (128)
**Fructose, ** ***g/day***	24.7 (11.6)	26.1 (12.3)	24.2 (11.5)	23.9 (10.6)
**Lactose, ** ***g/day***	21 (9)	21.7 (9.3)	21 (9.1)	20.3 (8.6)
**Calcium, ** ***mg/day***	1121.8 (285)	1163.5 (311)	1126.5 (284.1)	1078 (251.7)

### Purine-rich foods

**Figure 1:**
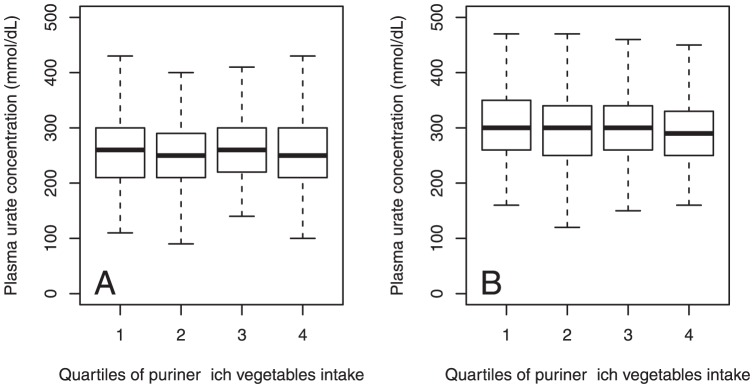
Plasma urate concentration in relation to quartiles of purine-rich vegetables intake for healthy (a) women and (b) men from Scotland, UK (1999–2006).

The mean number of daily servings of purine-rich vegetables was 1.9 (1.3) tablespoons. In a multivariate regression analysis the intake of purine-rich vegetables was not associated with urate concentration (*P* = 0.38) (**Figure 1** **,** **Table 2**). We found that meat intake was associated with urate concentration (*P* = 0.04), but not the intake of seafood in general (*P* = 0.69) or shellfish in particular (*P* = 0.28).

**Table 2:** The Association of Selected Food Products and Nutrients With Plasma Urate Concentration, in Healthy Adults From Scotland, UK.

	β for z-transformed variable	*P*
**Dairy**	−3.9	0.008
**Meat**	3.0	0.04
**Seafood**	0.6	0.69
**Fizzy drinks**	3.3	0.02
**Low-calorie fizzy drinks**	−1.8	0.25
**Pure fruit juice**	3.5	0.02
**Squashes**	2.8	0.06
**Sugar-sweetened beverages**	3.9	0.008
**Purine-rich vegetables**	−1.4	0.38
**Calcium**	−4.5	0.003
**Free fructose**	0.7	0.66

Regression Analysis was Adjusted for Age, Sex, BMI, Total Energy, Alcohol, Vitamin C and Coffee Intake (1999–2006).

### Sugar-sweetened beverages and fructose

Details on the reported intake of fizzy drinks, squashes, pure fruit juices and SSB are shown in **Table 3**. In a multivariate analysis, we found a significant positive association between SSB and urate (*P* = 0.008) (Table 2 and **Table 4**). The difference in urate concentration between participants that were drinking 2 or more SSB servings a day compared to those who did not drink any SSB was 13.7 (8.7) mmol/dL (*P* = 0.06). Fizzy drinks were also independently associated with urate (*P* = 0.02).

**Table 3:** Beverage Intake Among Healthy Adults From Scotland, UK (1999–2006).

Beverage^a^		N	mean (SD)
**fizzy**	all subjects	2076	0.08 (0.32)
	consumers	365 (17.6%)	0.47 (0.63)
**squashes**	all subjects	2076	0.27 (0.75)
	consumers	653 (31.5%)	0.87 (1.12)
**pure juice**	all subjects	2076	0.69 (0.86)
	consumers	1449 (69.8%)	0.99 (0.87)
**low-cal fizzy**	all subjects	2076	0.27 (0.71)
	consumers	707 (34.1%)	0.8 (1.03)
**SSB**	all subjects	2076	0.36 (0.82)
	consumers	863 (41.6%)	0.86 (1.1)

Mean beverage intake is shown for all individuals and for the subgroup that has reported taking specified beverage (“consumers”).

a servings/*day*.

**Table 4:** Urate Concentration in Relation to Sugar-Sweetened Beverage Intake and Fructose Intake, in Healthy Adults From Scotland, UK (1999–2006).

	sugar-sweetened beverages (SSB)				
	0 servings		≥2 servings		
	N	urate, mmol/L		urate, mmol/L	*P*
all	1188	281.25±71.64	94	294.89±81.73	0.06^a^
men	694	300.09±67.72	55	316.73±74.01	0.05
women	553	258.14±68.52	39	264.1±83.06	0.33

	free fructose^b^				
	bottom 25%		top 25%		
	N	urate, mmol/L	N	urate, mmol/L	*P*
all	504	285.16±69.19	512	273.16±72.51	0.004
men	282	298.19±66.11	291	303.64±72.49	0.19
women	222	258.2±66.46	221	251.99±63.96	0.16

	total fructose^c^				
	bottom 25%		top 25%		
	N	urate, mmol/L	N	urate, mmol/L	*P*
all	504	288±67.99	509	277.23±73.36	0.008
men	283	300.71±64.66	291	298.73±73.37	0.36
women	224	262.68±67.82	221	255.66±62.39	0.13

a the power to detect association has decreased due to individuals taking 1 or 2 servings being excluded.

b free fructose comprises a monosaccharide sugar fructose found in food and beverages.

c total fructose comprises free fructose plus fructose contained in the disaccharide sugar sucrose.

Among our participants free fructose intake was 24.7 g (11.6 g) and of total fructose intake was 55.9 g (18.0 g). Conversely, we did not find an inverse association between urate and free fructose intake in a multivariate analysis (*P* = 0.66) (Table 2). When comparing top and bottom intake groups, groups with higher intake had predominantly lower urate levels for both free and total fructose (Table 4). The percent of energy participants gained from free fructose was 3.8% (1.8%), range: 0.3% to 14.9% and from total fructose 8.6% (2.8%), range: 1.1% to 24.4% and these were also not associated with urate concentration (*P* = 0.72 and *P* = 0.78, respectively). There was no evidence of an association between glucose and sucrose intake (also found in abundance in SSB) with plasma urate (*P* = 0.50 and *P* = 0.98, respectively). The correlation coefficient between SSB and free fructose intake was 0.15 and total fructose 0.20.

### Dairy products

The mean intake of dairy products was 335.0 (192.5) g a day. Our results show an inverse association between dairy and urate concentration (Table 2). Skimmed milk and low-calorie yoghurt intake were associated with urate (beta = −4.4 mmol/dL per serving, *P* = 0.02 and beta = −11.7 mmol/dL per serving, *P* = 0.04), while the intake of semi-skimmed and full-fat milk, and low-fat and full-fat yoghurt were not (**Table 5**).

**Table 5:** The Association of Selected Dairy Products With Plasma Urate Concentration in Healthy Adults From Scotland, UK.

	β per serving	N consumers	*P*
youghurt, low calorie (125 mL)	−11.7	421	0.04
youghurt, low fat (125 mL)	−2.1	1001	0.63
youghurt, full fat (125 mL)	−6.2	255	0.57
milk, skimmed (150 mL)	−4.4	322	0.02
milk, semi-skimmed (150 mL)	−0.4	1398	0.76
milk, full fat (150 mL)	−1.3	466	0.51

Regression Analysis was Adjusted for Age, Sex, BMI, Total Energy, Alcohol, Vitamin C and Coffee Intake (1999–2006).

Milk and dairy products have a high content of calcium and lactose. The correlation coefficient between dairy and calcium intake was 0.76 and with lactose intake 0.88. Both nutrients (energy-adjusted and z-transformed) showed significant and inverse associations with urate, for calcium beta = −4.5 and *P* = 0.003 and for lactose: beta = −4.9 and *P* = 0.0007. When variables were not transformed, each 1 g of lactose was associated with 0.5 mmol/dL lower urate and each 1 mg of calcium with 0.02 mmol/dL lower plasma urate.

## Discussion

In this study, we found no association between purine-rich vegetables consumption and plasma urate. We found an association between plasma urate and SSB, but not with fructose intake. Dairy, calcium and lactose intakes were inversely associated with plasma urate.

### Measuring purines and their effect

Meat intake and diets rich in purines have been recognized as risk factors for gout since ancient times. More recently, experiments in animals and humans have confirmed the urate-raising effect of purines, and this is because uric acid is the end-product of purine metabolism in humans [12], [13]. However, estimating the impact of dietary purines on plasma urate concentration is problematic for several reasons. It is difficult to accurately measure purine content [10], [14], the bioavailability of purines from different dietary sources varies considerably [10] and can be altered by cooking [15], and not all purines affect urate equally [12]. Therefore, dietary recommendations should not be based solely on the average purine content of a dietary item, but it is critical that they also reflect the observed effect on plasma urate.

### Purine-rich vegetables

We did not find an association between the consumption of purine-rich vegetables and plasma urate. Our finding is in agreement with the recent study in 47,150 individuals that did not find an association between purine-rich vegetables and gout [10]. A decrease in plasma urate on a vegetarian diet [16] and when supplementing a diet with spinach have been shown previously [17]. Interestingly, reports of acute effects are different: 2–3 h after tofu or soybean intake plasma urate rose significantly [18], [19]. We did not find any studies that, in the long term, showed a significant positive association of purine-rich vegetable intake and urate (excluding the acute effects). Our results reinforce concerns about the validity of recommendations to restrict intake of purine-rich vegetables such as asparagus, cauliflower, beans, lentils and spinach in gout patients [20] or the appropriateness of general advice to restrict purine-rich foods, with no distinction between meat, seafood and purine-rich vegetables [5], [6]. Since advice to restrict purine-rich vegetables could be potentially harmful further epidemiological and experimental research is needed.

### SSB

The concurrent increase in the incidence of gout (US [2] and UK [1]) and consumption of soft drinks (US [21] and UK [22]) motivated the hypothesis that soft drinks might be a risk factor for gout. In support of this we found a significant association between the intake of sugar-sweetened beverages (SSB) and urate (increase of 4.8 mmol/dL per serving, *P = *0.008). Our findings replicate previously reported associations. A large study of 15,745 individuals found that those with higher SSB consumption were significantly more likely to be hyperuricemic [23] and another study comprising 14,761 individuals showed a significant association between SSB consumption and plasma urate [7].

We found a difference in the plasma urate concentration of 13.65 mmol/dL between individuals who do not consume SSB versus those who take ≥2 servings/day, similar to another study that argues such a difference is of clinical relevance and may considerably alter the risk of gout [9].

### Fructose

It is difficult to determine whether it is compound(s) present in SSB that cause the association with plasma urate or whether SSB is just a marker for an “unhealthy” diet and lifestyle. After numerous reports of the association between SSB and urate, fructose – the main sweetener in SSBs – was proposed as the causal agent. However, the evidence in support of this is conflicting.

Our data did not support the hypothesis that dietary fructose intake is positively associated with plasma urate concentration. We repeated this analysis with the proportion of total energy obtained from specific sugars, hypothesizing that it may be the relative rather than the absolute amount of fructose that is important, but the association remained not significant.

Evidence supporting the urate-increasing properties of fructose come from three sources. First, after absorption, the next step in fructose metabolism is phosphorylation in the liver. While phosphorylation of glucose is tightly regulated, ensuring that ATP is never depleted, phosphorylation of fructose is not regulated, which can lead to a decrease in intracellular phosphate. During this process, ATP is used and degraded to AMP and then to uric acid [24], [25]. Purine nucleotide depletion also accelerates purine synthesis, creating new precursors [26]. Second, observational evidence detected that the increase in the prevalence of gout [2] has been observed concurrently with the increase in fructose consumption [27], [28]. Thirdly, experimental studies in humans and animals show a short term rise in urate concentration following fructose ingestion or infusion [11], [29], [30], [31], [32]. However, fructose intervention studies are often criticized because the administered fructose is unnatural in the (a) amount and (b) the form of intake (often administered as a free fructose and/or parenterally). We found a mean intake of 24.7 g/day for free fructose and of 55.9 g/day for total fructose (previously reported 25 g/day and 54 g/day, respectively, in the UK [33]). It was also shown that >95% adults consume <100 g/day fructose (<20% of total energy), which means that results of intervention studies in humans that administer >100 g/day may have limited relevance for creating public health policies. The same applies to experimental studies in animals where >20% of energy was obtained from fructose.

The relationship between fructose and health outcomes may be dose-dependent and not linear. It is possible that fructose exhibits beneficial effects at low doses and adverse effects only at high doses [34]. Also, individuals consuming fructose at a high rate are likely to suffer inadequate intake of other micronutrients [34] and to consume other sugars in excess.

### Dairy

We have found a significant and inverse association between dairy intake and urate concentration, particularly with skimmed milk and low-calorie yoghurt, in accordance with numerous other studies [35], [36], [37], [38], [39], acutely after consumption [36], [37] and as a long-term effect [35], [38], [40], [41], [42], [43], [44], [45]. The absence of association with full-fat dairy products might arise from the counteracting effect of saturated fats [35]. The intake of dairy, especially low-fat products, was associated with a lower risk of gout [10].

Some of the possible mediators of urate-lowering effect of dairy are calcium, phosphorus, magnesium, lactalbumin, casein, lactose and orotic acid [42], [46]. Nutrients characteristic of dairy are highly correlated and experimental studies will be needed to clarify which compound is causally affecting urate concentration.

### Calcium

In the regression analysis, we found that a difference of 1000 mg/day of calcium intake corresponds to a 15.8 mmol/dL difference in urate concentration, similar to the 22 mmol/dL difference reported to be associated with calcium intake increased by 1200 mg/day [36]. However, an intervention study has demonstrated that calcium supplementation did not significantly affect urate (although baseline dietary calcium intake was associated with urate). This finding suggests that the protective effect of dairy products in preventing hyperuricemia and gout may not be mediated by dietary calcium [36].

In summary, the mechanism of the urate-lowering effect of dairy products is still not clear. Milk contains orotic acid which promotes renal urate excretion and could contribute to urate-lowering effects of dairy products. Casein and lactalbumin load have been shown to decrease plasma urate after two hour period [37], [39]. Finally, high dairy product consumption may reflect generally healthier diet and lifestyle.

### Current recommendations

European League Against Rheumatism (EULAR) recommendations postulate that patient education and appropriate lifestyle advice, including dietary advice, are core aspects of gout management [47]. However, dietary advice is given to only 30% of patients (range: 8–75%) [48] and available guidelines are often not up to date (**Table 6**). Interestingly, despite the weak evidence that restriction of purine-rich vegetables is beneficial, restriction is commonly recommended eg. [20]. In contrast, a higher intake of dairy is much less frequently recommended to control hyperuricemia [5], [6], although the evidence for urate-lowering effects of dairy products is much stronger.

**Table 6:** Dietary recommendations for gout patients.

Source	Published/Revised	Purine-rich Foods	Meat and Seafood	Purine-Rich Vegetables	Sugar-Sweetened Beverages	Dairy
**Jordan et al. ** [**5**]	May-07	limit	limit	-	-	increase skimmed and low-fat dairy
**UK Gout Society**	2009	limit	limit	limit mushrooms, asparagus, cauliflower, spinach, lentils, soya	-	increase dairy
**NHS**	Mar-10	limit	limit	limit asparagus, kindey beans, lima beans, lentils, spinach	-	-
**American College of Rheumatology**	Apr-10	-	limit	“it appears there is no need to avoid purine-rich vegetables”	-	“dairy might be good”
**National Institutes of Health US**	May-10	limit	limit	limit asparagus, dried beans and peas, mushrooms	-	-
**Arthritis Research UK**	Apr-11	limit	limit	-	avoid	increase skimmed milk
**US National Library of Medicine (by Mayo Clinic)**	Aug-11	limit	limit	-	-	increase skimmed or low-fat milk
**Arthritis Care UK**		limit	limit	-	-	-

### Limitations

Since this was an observational study, we did not have the ability to assess causality of reported associations. Many of the nutrients coming from the same sources are highly correlated, which makes the interpretation of results difficult. Study participants might have had a healthier lifestyle than the general population (participation bias). It is very complex to measure the typical diet of an individual. Dietary habits vary with season and age, and even the amount of nutrients in the specific product is rarely stable [28]. Although very practical, major limitation of FFQs is the inaccuracies that results from errors in reporting both frequency and serving size, and due to the incomplete list of food items. There is a tendency to under-report unhealthy eating habits and over-report healthy habits, possibly affecting our measure of fizzy drinks and squashes intake. It is difficult to accurately calculate the true lactose intake because of the recent and poorly recorded practice of adding lactose to different products. There are differences in food composition between UK and US as they may in part explain the differences between studies e.g. high-fructose corn syrup is less frequently used in Europe.

Information on renal function, diabetes and hypertension and medication was not available, and these might have affected the results e.g. because diuretics can lead to an increase in urate [49]. Endogenous purines are an important additional source of purines but estimates of their content in the diet is not possible.

### Conclusion

The lack of a significant association between purine-rich vegetable intake and plasma urate challenges the appropriateness of recommendations to restrict purine-rich vegetables in hyperuricemic individuals and gout patients. The abundant evidence supporting the inverse association between plasma urate concentration and dairy consumption should be reflected in dietary guidelines aiming to lower plasma urate. Our results do not support the hypothesis that fructose is the causal agent underlying the association between SSB and urate. Further studies are necessary to establish which nutrients and food products causally affect plasma urate. This information is necessary for providing accurate and comprehensive dietary guidelines for gout patients and hyperuricemic individuals.

## Materials and Methods

The study population comprised 2,076 adults included as healthy controls in a population-based case control study of colorectal cancer in 1999–2006. This study has been described in detail elsewhere [50]. Controls included in this study were identified throughout Scotland matched for age, sex and region of residence with colorectal cancer cases then drawn randomly from the Community Health Index, a population register with high completeness, and invited by their general practitioner to take part. We did not include the colorectal cancer cases, because of a possible change in the diet and lifestyle after diagnosis and other changes related to treatment and disease. All participants gave written informed consent. The participation rate among controls was 57%. Approval for the study was obtained from MultiCentre Research Ethics Committee for Scotland and Local Research Ethics committee.

### Data collection and samples

Participants completed a semi-quantitative food frequency questionnaire (FFQ, Scottish Collaborative Group FFQ, Version 6.41) which consisted of 150 items, and the individuals were asked to describe the amount and frequency of each food on the list they have eaten a year before recruitment. The FFQ was used to calculate each individual's intake of food items (eg. dairy products, meat and SSB) and nutrients (eg. fructose, calcium). The main characteristics and validity of the FFQ have been reported previously [50], [51]. All intake data are reported as daily intakes, either as the number of servings (food products) or by weight (nutrients). The following definitions were adopted throughout the study:

- “Dairy” is a measure of dairy product consumption. It includes: milk (skimmed, semi-skimmed and whole), yoghurt, cream and cheese and is reported as the total number of 25 g portions of any of the above.
- “Meat” represents the number of meat servings (50–110 g), including: mince or meat sauce (1 serving = 2 tablespoons), sausages (1 sausage), burgers (1 burger), beef (2 tablespoons, 2 slices or 1 steak), pork and lamb (2 tablespoons, 2 slices or 1 chop), chicken and turkey (1 wing or thigh, ½ breast or 2 slices), bacon and gammon (1 medium slice), liver and liver products (1 serving), haggis or black pudding (2 tablespoons or 1 slice), meat pies pasties and sausage roll (1 individual pie or 1 roll), cold meats, salami and continental sausage (1 slice).
- “Seafood” represents the number of fish and shellfish servings (50–150 g), including: fish fingers (1 finger), white and oily fish (1 small fillet), fish cakes and pies (1 cake or 2 tablespoons), sardines and pilchards (2 pieces), tuna (1 table spoon), prawns, crab, mussels, oysters, cockles and scallops (1 table spoon without shell). “Shellfish” represents the number of servings, including only prawns, crab, mussels, oysters, cockles and scallops.
- “Purine-rich vegetables” is expressed as the number of servings (1 tablespoon) of purine-rich vegetables, including: cauliflower, spinach, peas, beans, and lentil.
- “Sugar-sweetened beverages” (SSB) is the number of daily servings of squashes (medium glass) and fizzy drinks (can). “Squashes” is the number of medium-sized glasses of blackcurrant and other types of fruit squashes.

Using an in-house calculation program, nutrients were calculated from the consumption frequencies of a specified portion size for each item from the FFQ, adjusted for total energy intake [52]. The fructose and lactose intakes were estimated using individual sugar composition data for foods included in the FFQ. These were taken from the Food Standards Agency (2009) McCance and Widdowson's composition of foods integrated data set [53]. Free fructose represents all fructose from dietary sources, and total fructose also includes fructose released after the hydrolysis of sucrose. Validation of the questionnaire indicated that total sugars were in reasonable agreement with estimates from diet diaries [51] with a higher level of agreement in females (r = 0.72) than males (r = 0.41). Alcohol intake was calculated from the reported amount and type of beverages consumed.

Participants were also asked to give details about supplement intake and nutrient information was collected from the manufacturer's product information. If a compound was found both in foods and supplements, their total was used in the analysis (e.g. calcium).

### Blood sampling, preparation and plasma urate assay

Blood samples were sent to research centre and stored at −40 degrees usually within 24 hours of sampling (always within 72 h). All plasma urate measured at a single point in time and in one laboratory. Diet prior to sampling was not controlled. Urate concentration was available for 2037 participants. Hyperuricaemia was defined by cut-off values of >415 mmol/dL for men and >340 mmol/dL for women [7], [54].

### Statistical analysis

Statistical analysis was performed using R [55]. In the multivariate regression analysis, the significance of the association between plasma urate concentration and selected nutrients and dietary items was assessed. The regression model was adjusted for relevant variables: age, sex, BMI, total energy, alcohol, vitamin C and coffee intake [7], [54]. In some cases, to enable comparison of effect sizes (beta-coefficients) of variables that were reported in different measuring units, variables were transformed to standard normal distribution (z-transformed). Mean and standard deviation are reported as: mean (SD).
